# Predictors for elevation of Intraocular Pressure (IOP) on glaucoma patients; a retrospective cohort study design

**DOI:** 10.1186/s12886-022-02431-w

**Published:** 2022-06-07

**Authors:** Getasew Birhanu, Awoke Seyoum Tegegne

**Affiliations:** 1 Department of Statistics, Dilla University, Dilla, Ethiopia; 2 Department of Statistics, Bahir Dar University, Bahir Dar, Ethiopia

**Keywords:** Glaucoma, Elevation of IOP, Extreme blood pressure, Diabetic, Hypertensive, Socio-demographic characteristics, Individual behaviour, Clinical factors

## Abstract

**Objective:**

Because of the increase in the number of cases, currently, glaucoma is a significant public health issue that it leads to optic nerve damage and vision loss. High Intraocular Pressure reading indicates that the treatment given to a glaucoma patient is not sufficient/ adequate. Hence, the elevation of intraocular pressure is one of the indicators that, the therapy given to glaucoma patients under treatment is inadequate. Therefore, the main objective of the current study was to investigate predictors for the variation of elevation of IOP readings on glaucoma patients.

**Materials and methods:**

A retrospective cohort study design was conducted on 1254 glaucoma patients, whose followed-ups were from September 2015 to August 2016 at Felege Hiwot Teaching and Specialized Hospital, North West Ethiopia. Data analysis was conducted using Statistical Analysis of Systems (SAS) software version 9.2 and AMOS software. The parameter estimation was conducted using the maximum likelihood estimation technique.

**Results:**

Main effects like age (*β* = 0.01, *t*-value = 0.15, *p*-value = 0.018), patients with normal blood pressure (*β* = -3.35, *t*-value = -2.28, *p*-value = 0.0263), patients without diabetics (*β* = -3.79, *t*-value = -2.47, *p*-value = 0.014), visiting times (*β* = -6.00, *t*-value = -5.02, *p*-value = 0.0001), farmer glaucoma patients (*β* = -6.04, *t*-value = 3.87, *p*-value = 0.0001) had significant and indirect effect for the variation of elevation of IOP on glaucoma patients. Interaction effects like visiting time with existence of diabetes, visiting time with cataract surgery significantly effected on the variable of interest. Hence, both main and interaction effects had significant effects on the variable of interest.

This study had identified socio-demographic characteristics, personal/individual behaviors, and clinical factors for the variation of elevation of IOP. The findings, in the current investigation, help health staff to conduct health-related education for awareness creation. Health-related education, about the progression of glaucoma, should be conducted on patients.

**Supplementary information:**

The online version contains supplementary material available at 10.1186/s12886-022-02431-w.

## Background

Glaucoma is a chronic progressive disease and a leading cause of blindness, affecting more than 70 million people worldwide [1]. The number of glaucoma patients is increasing with the world population growth and it is a chronic eye disease of individuals [2]. Glaucoma is branded by a progressive loss of retinal ganglion cells (RGCs) [3]. It leads to optic nerve damage and vision loss [4].

The progression of glaucoma patients under treatment can be detected using different techniques. Investigation of the association between cup-to-disc ratio (CDR) and estimates of retinal ganglion cell (RGC) number is means of assessing the progression of glaucoma disease [5]. Evaluation of the optic disc is another indicator for progression of glaucoma disease [6]. The optic disc is a small blind spot on the surface of the retina and is composed of RGC axons, which at the surface of the disc bend highly to exit the eye through the lamina cribrosa [7].

The rim-to-disc ratio (RDR) also accurately segments the optic disc and optic cup and then computes the RDR based on which it is possible to differentiate a normal fundus from a glaucomatous one [8]. Circumpapillary retinal nerve fiber layer (cRNFL) thickness is also an indicator of the progression of glaucomatous damage. Hence, cRNFL thickness is used to detect progression of glaucoma disease [9].

Structural properties (e.g. presence of the optic nerve head and thickness of the retinal nerve fiber layer and ganglion cell complex), and functional characteristics (e.g. visual field sensitivity) of the visual pathway are some of the other techniques of assessing the progression of glaucoma [10].

The assessment for progression of glaucoma disease also consists of event-based or trend-based. In event-based analyses, a criterion for progression of glaucoma disease should be compared with baseline [11]. This could be happened for each parameter, considering on a statistical calculation of confidence interval for test-retest variability [11]. Event analyses indicate progression of glaucoma when a measurement exceeds a predetermined criterion for progression [12]. On the other way, trend-based analyses use linear or other forms of regression analyses to evaluate rates of progression in parameters [13].

Another method of detecting the progression of glaucoma disease is based on the elevation of IOP [14]. Hence, the elevated IOP on glaucoma patient is an indicator that the therapy given for the patients is not adequate and need further interventions [15]. When the IOP reading is greater than 21 mm of Hg, the IOP is said to be elevated [16].

Among the above different indicators for progression of glaucoma disease, in this research, more emphasis was given for elevation of IOP, in which many of the previous researchers didn’t give attention for this parameter in the study area, Ethiopia [16].

A prevalence study for East, Central, and Southern Africa indicates that about 10,000 people are affected by glaucoma with an annual incidence rate of 400 new cases per million populations [17, 18]. The prevalence of glaucoma in Sub-Saharan Africa is also very high and thus has been considered as a major public health issue for the region [19].

Ethiopia is one of the Sub-Saharan Africa in which more than 80 million people are affected by glaucoma and 1.4% of them are under treatment [20]. In Ethiopia including the catchment area of Felege Hiwot Teaching and Specialized Hospital, glaucoma patients under treatment, who did not properly adhere to their prescribed medication by the health staff, have high IOP elevation reading, and glaucoma in such case caused irreversible blindness for an estimated number of 62,000 people [21]. The treatment of glaucoma is also given to prevent the poor vision-related quality of life [22]. Hence, elevation of IOP is used to assess a glaucoma progression (treatment outcome) such that the higher elevation of IOP indicates that the therapy given a patient is inadequate or patients did not adhere the prescribed medication properly.

Identifying socio-demographic, individual behavior and clinical risk factors affecting elevation of IOP is crucial for detecting progression of glaucoma patients under treatment and to understanding the path physiology of glaucoma [23]. Previously, a research was also conducted on how the progression of glaucoma is detected using a rate of visual field and rate of structural loss without considering how the elevation of IOP reading used to indicate the progression of glaucoma [24]. This indicates that, a glaucoma patient with normal IOP reading ( < = 25 mm of HG) indicates that a therapy given to a glaucoma patient was sufficient/adequate and a patient with an elevated IOP(> 25 mm HG) indicates that a therapy given to glaucoma patients was inadequate [25].

Another study declared that a larger cup-to-disc ratio is used to assess for the progression of glaucoma [26]. Population-based epidemiologic studies also stated that there is a strong association between the low elevation of IOP and open-angled glaucoma prevalence and incidences [27]. Clinical studies, on the other hand, reported that there is a similar association between the elevation of IOP and open-angled glaucoma progression [28].

Recently, an investigation was conducted on the effects of elevation of IOP on the lamina cribrosa (LC) in vivo on rhesus monkeys [28, 29]. The investigators in this regard manipulated the IOP while imaging the LC in multiple IOP combinations and measured different structural features [29].

Findings from the previous research indicate that there are inconsistencies between different investigations [30]. The previous studies did not investigate the interaction effects between covariates for the elevation of IOP on glaucoma patients [31]. Therefore, the objective of this study was to investigate predictors for the variation of elevation of IOP readings on glaucoma patients. This study also aimed to assess whether the results obtained in developed countries also worked in the study area. To the best of our knowledge, there is a scarcity of studies investigated previously about factors for the variation of elevation of IOP reading on glaucoma patients in the study area.

## Materials and methods

### Study design and settings

 Institution based a retrospective cohort study design was employed among glaucoma patients at Felege Hiwot Teaching and Specialized Hospital, Amhara region, north-west Ethiopia. The study was conducted from September 2015 to August 2016. The ophthalmic clinic at the hospital is one of the largest in the Amhara region, which has been serving more than 400 patients per week.

### Sample size and sampling technique

A sampling frame was prepared in collaboration with health staff in the ophthalmic clinic in the hospital. A stratified random sampling technique was applied in the sample unit selection procedure, considering their residential areas as strata. The sample size was determined using single proportion formula with the following assumptions: estimated proportion of glaucoma patients 60% (𝑝 = 0.60) (18), 95% CI: (𝑍_𝛼/2_ = 1.96), and a 5% margin of error (e = 0.05). Hence, the final sample size was therefore 1250.

### Data collection tools and procedures

The information was first observed and discussed with health care service providers at the ophthalmic clinic in the hospital. Data were extracted using a data extraction format developed by the investigators in consultation with health care service providers. All important information was collected by the health care service providers after theoretical and practical orientations. Charts of patients were retrieved using the patients’ registration card number which was found in the electronic database system.

### Source of data

 Secondary data were obtained from charts of randomly selected 1250 glaucoma patients at Felege-Hiwot Teaching and Specialized Hospital, North-west Ethiopia.

### Data quality

The quality of the data was controlled by data controllers from the ophthalmic clinic. Data collectors had been oriented about the variables included under investigation.

### Inclusion and exclusion criterion

 Glaucoma patients who had at least three follow-up visits for treatment and patients who had full medical records were included under the given investigation. On the other hand, patients who had glaucoma surgery may bias the result and were excluded from this study. Patients, who lacked records, regarding the variables under investigation, were also excluded from the current investigation.

**Fig. 1:**
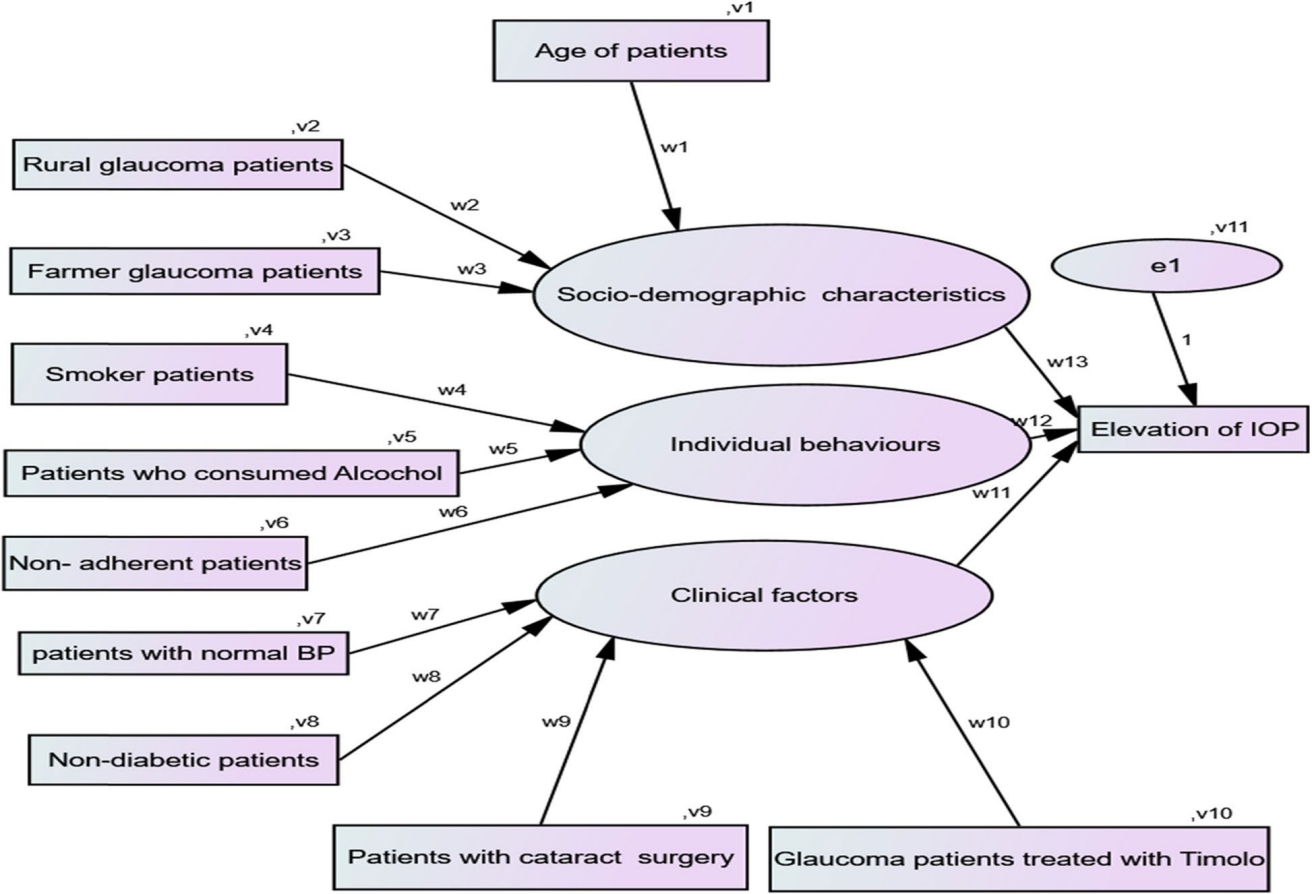
Path analysis for the relationship between endogenous and exogenous variables

In the current investigation, the elevation of IOP reading in mm of Hg for glaucoma patients was considered as the response variable which is continuous in nature. The explanatory variables which might affect for the elevation of IOP reading on glaucoma patients were categorized as socio-, demographic characteristics, individual behaviors, and clinical factors. The predictor variables under current investigation were age in years, place of residence (urban, rural), occupation type (farmer, others), medication adherence(adherent, non-adherent), smoking status (yes, no), alcohol intake (yes, no), the existence of extreme blood pressures (yes, no), the existence of diabetes (yes, no), availability of cataract surgery (yes, no), and type of treatment (Timolol, Pilocarpine, Timolol with Diamox, Timolol with Pilocarpine, Timolol with Pilocarpine and Diamox). The path analysis which shows the relationship between exogenous and endogenous variables in the current investigation is indicated in Fig. 1.

### Variables understudy

**Fig. 2:**
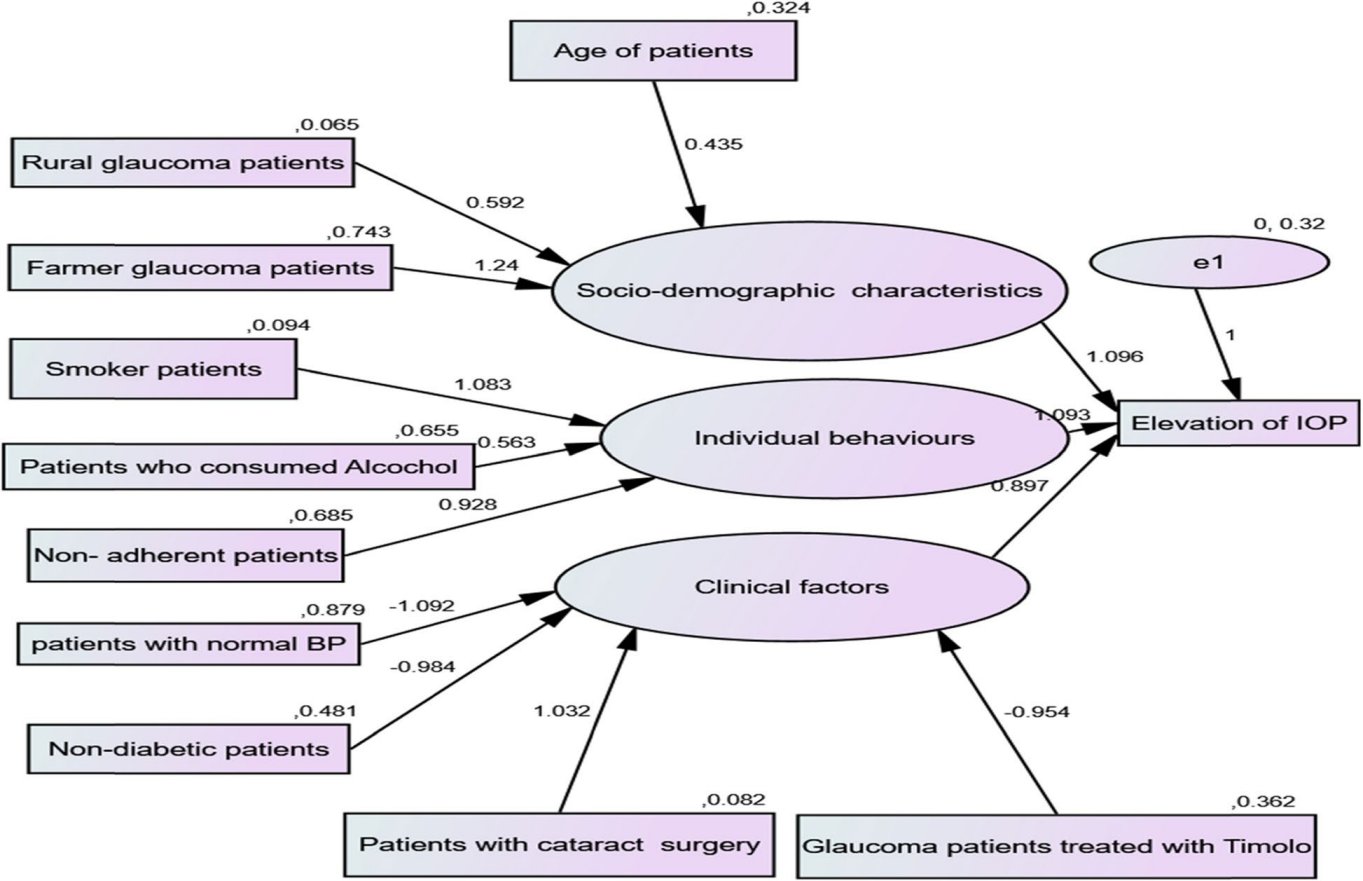
Path analysis framework of covariates along with calculated values of weighted regression coefficients and their variances

The relationship between dependent/endogenous and independent/exogenous variables in Fig. 1 shows that w_1_-w_13_ represented the weighted regression coefficients and v_1_-v_11_ represented the variance of predicted variables. The weighted regression coefficients might be either positive or negative. The negative coefficient indicates that exogenous/predictor variables had a negative association with endogenous/dependent variables whereas, a positive value of the weighted coefficient represents that the predictor and dependent variable had a positive association between them. In the current investigation, socio-demographic characteristics, individual behaviors, and clinical factors are latent predictors for the elevation of IOP in glaucoma patients. The path analysis, in Fig. 1, shows that there were direct and indirect effects of predictors on the variable of interest. Hence, the age of patients, residence area, and occupation type of individual had a direct effect on socio-demographic characteristics and an indirect effect on the elevation of IOP readings. Similarly, smoking status, alcohol intake, and medication adherence belong to individual behavior and further affected the elevation of IOP readings indirectly. Finally, the Health status of patients (existence of normal BP, non-existence of diabetes, and cataract surgery) and treatment type were categorized as clinical factors that affected the elevation of IOP reading indirectly. The estimated values of regression weighted coefficients and the variance of estimated parameters are indicated in Fig. 2 (in the Results section).

Clinical factors, individual behaviors, and socio-demographic characteristics (variables under circles) are latent/unobserved variables expressed interims of observed variables indicated by the arrows (Refer to Fig. 1). Elevation of IOP is the dependent variable and all variables under rectangles are predictor variables.

### Data analyses

Data were entered and analyzed using the Statistical Analysis of Systems (SAS) software version9.6. SAS software is used for the analysis of longitudinal and repeated measures either retrospective or prospective data. In data analysis, both descriptive and inferential statistics were carried out to describe variables and to infer about the entire glaucoma patients based on the information obtained from the selected samples. Multivariable logistic regression analysis was used to identify predictors for the elevation of IOP. The path analysis was constructed using AMOS software. The estimation of weighted regression coefficients and variance components were also calculated using AMOS software.

### Diagnosis and treatments performed under investigation

First, the appearance of the optic nerve head and thickness of the retinal nerve fiber layer and ganglion cell complex and functional characteristics of glaucoma patients were tested and evaluated for all patients. Similarly, the elevation of IOP was assessed for glaucoma patients who came for treatment purposes. The visual field sensitivity of the visual pathway of patients was also assessed critically. Regular follow-up assessments were then performed throughout the patients’ visit to the hospital. Hence, the elevation of IOP for glaucoma patients was assessed at each visiting time of patients.

AGIS severity scoring system and the CIGTS visual field classification were used to assess the progression of the glaucoma disease. The AGIS trial was planned to investigate progression for advanced glaucoma patients who are not controlled by medications and are in need of surgery.

The CIGTS trial was designed to regulate whether the better initial treatment for newly diagnosed glaucoma patients was medications or immediate filtration surgery [32]. Hence, both the event analysis (guided progression analysis, early manifest glaucoma trial, progression classification systems) and trend analysis (global indices, point-wise linear regression, clusters) were also designed.

### Models used for parameter estimation

A linear mixed effect model (LMM) is a parametric model for longitudinal or repeated measures data that quantifies the relationships between a continuous dependent variable and various predictor variables. An LMM may include both fixed-effect parameters associated with one or more continuous or categorical covariates and random effects associated with one or more random factors. Fixed-effect parameters describe the relationships of the covariates to the dependent variable for the entire population; random- factors are specific to subjects within a population. Consequently, random effects are directly used in modeling the random variation in the dependent variable at different levels of the data [33].

The linear mixed-effects model assumes that the observations follow a linear regression where some of the regression parameters are fixed or the same for all subjects, while other parameters are random or specific to each subject [33, 34]. Correlated continuous outcomes are treated as fixed effects. The general form of the linear mixed-effects model after combining the two stages is approximately normal [33–36].

### Methods of parameter estimation in LMM

The maximum likelihood estimation (MLE) was used for parameter estimation. These methods are based on maximizing the marginal likelihood function, which is a mathematical expression that describes the joint probability of obtaining the data expressed as a function of the parameters.

### Model adequacy and selection procedure

 For the purpose of model selection, AIC and other information criteria were used. After the final model selection, the model was refitted using restricted maximum likelihood (REML) estimation methods. Two models, the full/saturated model, and the reduced models were compared using REML. Likelihood ratio tests under REML were used to assess the importance of the covariance parameter estimates [37].

## Results

The baseline characteristics of elevation of IOP in glaucoma patients and baseline characteristics are indicated in Table 1. In Table 1, it is indicated that among 1254 study units, 432 (34.5%) were females, 672(53.6%) were urban residents, 594(47.4%) took alcohol, 366 (29.2%) had abnormal blood pressure, 348(27.8%) were diabetic patients, 492(39.2%) had no other eye or ocular problem, 180 (14.4%) had cataract surgery. Out of the participants, 33.5% were farmers and the rest were others (daily workers, government workers, and housewives). Among the glaucoma patients under treatment, 248 (19.8%) had an elevated IOP(whose IOP level > normal level), 54 (4.3%) had corneal scar problem and 120 (9.6%) had eye trauma problems, The treatment type category indicates that 184(13.9%) cases were treated with Timolol, 96 (7.7%) cases were treated with Pilocarpine, 54 (4.3%) cases were treated with both Timolol with Diamox, 618(49.3%) cases were treated with both Timolol with Pilocarpine, 312(28.9%) cases were treated with Timolol & Pilocarpine and Diamox. Among all glaucoma patients under treatment, 44% were non-adherent to medication adherence.

**Table 1:** Baseline characteristics of elevation of IOP on glaucoma patients

Categorical variable	Category	n	%
Place of residence	Urban	672	53.59
	Rural	582	46.41
Occupation	Farmer	420	33.49
	Others	834	66.51
Smoking status	No	1182	94.26
	Yes	72	5.74
Alcohol intake	No	660	52.63
	Yes	594	47.37
Having abnormal blood pressure	No	888	70.81
	Yes	366	29.19
Have diabetes	No	906	72.25
	Yes	348	27.75
Existence of Cataract surgery	No	1074	84.65
	Yes	180	14.35
Existence of elevated IOP (> the normal range)	No	1006	81.20
	Yes	248	19.80
Existence of eye trauma	No	1134	90.43
	yes	120	9.57
Existence of Corneal scar	No	1200	93.69
	Yes	54	4.31
Glaucoma patients free from other ocular problem	Not free	762	40.77
	Free	492	39.23
Having cataract surgery	No	1128	89.95
	Yes	126	10.05
Medication adherence	Adherent	702	55.98
	Non-adherent	552	44.02
Types of treatment	Timolol	174	13.88
	Pilocarpine	96	7.66
	Timolol with Diamox	54	4.31
	Timolol with Pilocarpine	618	49.28
	Timolol, Pilocarpine and Diamox	312	24.88

The descriptive results like mean and standard deviation of IOP for each category are indicated in Table 2

The descriptive results in Table 2 indicate that the average IOP level of rural patients was greater as compared to urban. From the occupation type, farmers had a greater average elevation of IOP as compared to the other groups. The average elevation reading of IOP for patients who had cataract surgery was greater than those patients who had no cataract surgery. From the treatment type, patients who had been treated with Timolol, Pilocarpine, and Diamox had a more average level of IOP as compared to other treatment categories. The average level of IOP for diabetic patients was greater than those patients without diabetes. Glaucoma patients who had taken alcohol had a more average level of IOP as compared to patients who did not take alcohol. Finally, the average IOP level of glaucoma patients who were not free from any other ocular problem was greater than those of patients free from other ocular problems. The path analysis with the calculated value of weighted regression coefficients and the estimated value of variances are indicated in Fig. 2.

**Table 2:** Mean and standard deviation of IOP on glaucoma patients for categorical variables

Categorical variable	level	IOP readings	
		Mean	Std Deviation
Gender	Female(f)	28.45	10.25
	Male(m)	34.80	14.02
Place of residence	Urban	28.32	9.42
	Rural	37.56	15.07
Occupation	Civil servant	26.01	8.26
	Merchant	31.48	9.20
	Farmer	40.12	15.67
	Daily worker	31.44	11.54
	Other	29.01	10.07
Smoking status	No	32.76	13.51
	Yes	30.14	5.32
Alcohol intake	No	31.35	12.28
	Yes	34.01	14.02
Having blood pressure	No	31.36	12.99
	Yes	35.64	13.20
Have diabetes	No	30.81	12.41
	Yes	37.30	14.01
Existence of Cataract	No	28.62	13.54
	Yes	42.15	16.22
Existence of eye trauma	No	27.34	9.81
	yes	31.33	9.08
Existence of Corneal scar	No	19.76	8.19
	Yes	30.42	9.25
Having cataract Surgery	No	32.03	13.02
	Yes	37.77	13.63
Types of treatment	Timolol	25.92	9.76
	Pilocarpine	27.56	9.87
	Timolol with Diamox	28.63	9.37
	Timolol with Pilocarpine	32.79	13.13
	Timolol, Pilocarpine and Diamox	38.23	13.94

The results of path analysis in Fig. 2 indicate that age of patients, rural patients, patients who are working on farming directly affected the socio-demographic variables and indirectly affected the elevation of IOP. Smoker glaucoma patients, glaucoma patients who consumed alcohol, and non-adherent glaucoma patients directly affected individual behaviors and indirectly affected the elevation of IOP. Similarly, patients with normal blood pressure, non-diabetic glaucoma patients, cataract surgery glaucoma patients, and patients treated with Timolol directly affected the clinical factors and indirectly affected the elevation of IOP. On the other hand, socio-demographic variables, individual behaviors, and clinical factors are affected directly by the elevation of IOP in the current investigation. The statistical parameter estimation with significance values (*p*-values) of the estimated value is indicated in Table 3.

**Table 3:** Parameter estimation for fixed effect result using linear mixed effect model

Effect	Estimates (*β* values)		Standard Error	*t*- value	Pr >|t|
Intercept	30.80		6.20	4.94	< 0.001*
Visiting time/ follow-up times	-6.00		1.20	-5.02	< 0.001*
Gender (Ref = Male)					
Female	-3.30		1.44	-2.30	0.321
Age	0.01		0.07	0.15	0.018*
Occupation (Ref = Other)					
Farmer	6.04		1.56	3.87	0.0001*
Alcohol intake (Ref = Yes)					
No	-1.18		1.23	-0.96	0.0381*
Bp (Ref = yes)					
No	-3.35		1.50	-2.23	0.026*
Diabetes (Ref = Yes)					
No	-3.79		1.54	-2.47	0.0139*
Cataract surgery (Ref.=no)					
Yes	2.79		1.24	1.27	0.0146*
Duration of treat (Ref = Short)					
Long	-3.51		1.87	-1.88	0.060
Medium	-1.68		1.77	-0.95	0.3418
Type of treatment (Ref = Timolol)					
Timolol + Pilocarpine	4.74		1.94	2.44	0.015*
Timolol + Diamox + Pilocarpine	5.96		2.25	2.65	0.028*
Existence of eye trauma(Ref.=no)					
Yes		2.71	0.72	3.73	0.002*
Existence of Corneal scar(Ref.=no)					
Yes		1.84	0.72	2.73	0.012*
Follow-up visits ^a^diabetes (Ref = yes)					
Follow-up visits ^a^no	0.40		0.29	1.35	0.018*
Follow-up visits ^a^cat surgery (Ref = yes)					
Follow-up visits ^a^no	2.71		0.72	3.73	0.002*

^a^stands for statistical significant variables

The different covariance-structures were considered and compared each other based on values of -2Log Likelihood, AIC, AICC, and BIC, with the assumption of the smaller is the better principle. The comparison of covariance-structures is indicated in Table 4.

**Table 4:** The comparison of covariance-structures

Fit Statistics				
	UN	AR(1)	TOEP	CS
-2 Res log likelihood	**8925.5**	8927.5	8927.5	8927.5
AIC	**9003.5**	9005.5	9003.5	9005.5
AICC	**9005.6**	9008.0	9005.9	9008.0
BIC	**9130.3**	9135.8	9130.5	9135.8

Hence, in the current study, the unstructured covariance-structure had smaller − 2Log Likelihood, AIC, AICC, and BIC of the fitted model and it was in favor of other variance-covariance structures. Based on the final selected model, a linear mixed effect model for parameter estimation was used to infer for the entire patient. The parameter estimates of the final model are summarized in Table 3.

As it is indicated in Table 3, the main effects; age, the existence of abnormal blood pressure, diabetics, visiting times, occupation type, alcohol intake status, and cataract surgery had significant effects on the elevation of IOP on glaucoma patients.

For a unit increase of follow-up visits, the expected elevation of IOP was decreased by 6 mm of Hg (*β* = -6.00, *t*-value =-5.02, *p*-value < 0.0001) keeping the other variables constant.

The expected elevation of IOP of glaucoma patients, for occupation type farming, was increased by 6.03 mm of Hg (*β* = 6.03, *t*-value = 3.87, *p*-value = 0.0001) as compared to patients with other occupation types, keeping the other variables constant.

As the age of glaucoma patients increased by one year, the expected elevation of IOP was increased by 0.01 mm of Hg (*β* = 0.01, *t*-value = 0.15, *p*-value = 0.018), keeping the other variables constant.

The expected elevation of IOP on glaucoma patients’ normal blood pressure was decreased by 3.36 mm of Hg (*β* = -3.36, *t*-value = -2.23, *p*-value = 0.0263) as compared to hypertensive glaucoma patients, keeping the other variables constant.

Similarly, the expected elevation of IOP for non-diabetic glaucoma patients was decreased by 3.79 mm of Hg (*β* = -3.79, *t*-value = -2.47, *p*-value = 0.0139) as compared to diabetic patients, keeping the other variables constant.

The expected elevation of IOP of glaucoma patients who had cataract surgery was increased by 2.76 mm of Hg (*β* = 2.76, *t*-value = 1.27, *p*-value = 0.0146), keeping the other variables constant.

Alcohol intake had also its own significant effect on the elevation of IOP in glaucoma patients. Hence, the expected elevation of IOP for patients who did not consume alcohol was decreased by 1.18 mm of Hg (*β* = -1.18, *t*-value = - 0.93, *p*-value = 0.0381), keeping the other things constant.

The treatment type, given for glaucoma patients, also affected the elevation of IOP; hence patients who took different types of treatments had more average elevation IOP as compared to those patients who took only one type of treatment.

The expected IOP elevation reading for glaucoma patients with eye traumas was increased by 2.71 as compared to those glaucoma patients without eye trauma (*β* = 2.71, *t*-value = 3.73, *p*-value = 0.002) keeping the other covariates constant.

In addition to main effects, the current investigation had also significant interaction effects. The two significant interactions effects are indicated below.

### Interaction effect between cataract surgery and follow-up visits

As number of follow-ups increased by one unit, the decreasing rate of average elevation of IOP for glaucoma patients without cataract surgery was greater by 2.7 as compared to patients with cataract surgery (*β* = 2.70, *t*-value = 3,73, *p*-value = 0.0002).

### Interaction effect between diabetic status and follow-up visits

 Table 3 indicates that as visiting time of a patient increased by one unit, the rate of decrease of expected elevation of IOP for glaucoma patients without diabetes was greater by 0.40 (*β* = 0.40, *t*-value = 1.35, *p*-value = 0.0178) as compared to patients with diabetes.

## Discussions

For glaucoma patients who were treated according to the schedule given by the care providers, the average elevation of IOP reduced with an increase of follow-up visits; however, the rate of reduction was different from one group to another.

The expected elevation of IOP of farmer glaucoma patients is greater than the other work types. The possible reason for this may be farmers come to treatment after they lost their eye vision as compared to other groups. Most of the time, farmers are exposed to dust particles, smoke (dangerous to the eye) and they may not take treatments properly. The nature of their work also had a significant effect on the elevation of IOP not to be reduced easily with treatment. This result is consistent with previously published articles which are stated as the higher expected elevation of IOP for farmers is not surprising, since farmers are more exposed to trachoma, glaucoma, etc. [28].

Treatment regimen complexity has certain effects for the elevation of IOP which means taking different treatment regimens at the same time may have the side effects of one to another. Hence, the type of treatment given for glaucoma patients also affects the elevation of IOP in glaucoma patients. This result is the same as that of previously conducted research [29].

Adherent patients have treatment improvements as compared to non-adherent patients. Non-adherent patients have more elevated IOP than adherent ones. Adherent patients believe that glaucoma would affect their eyesight in the future time and they believe that glaucoma medication would prevent vision loss. This result is consistent with two of the previous results [29, 38]. As information obtained in previous research, a challenge related to eye drop administration is one of the concerns for the patients to be non-adherent [38]. Hence, it needs social support for the treatment to be effective.

The expected elevation of IOP of glaucoma patients with abnormal blood pressure is greater than those patients with normal blood pressure. The result indicates that the therapy given for glaucoma patients with elevated IOP patients having abnormal blood pressure is inadequate as compared to patients with normal blood pressure. This result is supported by one of the previous studies [39].

Similarly, the expected elevation of IOP reading of glaucoma patients having diabetes is greater than those patients without diabetes. The result indicates that the therapy given to glaucoma patients having diabetes is inadequate as compared to non-diabetic patients. This result is consistent with one of the previous studies [38].

Glaucoma patients with cataract surgery have a higher elevation of IOP reading as compared to those patients who had no cataract surgery. Hence, the therapy given for glaucoma patients having cataract surgery is inadequate as compared to patients without cataract surgery. This result is consistent with another previous study [40] which stated that the therapy given to glaucoma patients after cataract surgery is inadequate and needs further intervention compared to patients treated without cataract surgery. On the other hand, it is not uncommon for glaucoma patients who already have a compromised trabecular meshwork to experience pressure spikes after cataract surgery [41]. Removing the cataract lens also reduces the production of PEX material [17, 33, 34] that may produce obstruction of the collector openings. There is a similar result from the previous research [17, 33, 34].

Glaucoma patients under treatment, who have also ocular infection, eye trauma, and corneal scar, have more elevation of IOP readings which further indicates that inadequate therapy is given for such patients. The result obtained in this regard is consistent with one of the previous researches[38].

Glaucoma patients under treatment who are drinking alcohol have a higher elevation of IOP readings as compared to those patients treated with the disease without alcohol. Hence, alcohol has a significant effect on the elevation of IOP and this indicates that the therapy given for such patients is inadequate and the glaucoma may leads to irreversible blindness of the eye [33–36].

Naturally, it is known that as age increases, the power of the eye vision of human beings becomes weak. The current investigation also assured that as age increased the elevation of IOP reading for glaucoma patients increased and this further leads to the fact that aged glaucoma patients may not easily cure from the disease and such people need further and continuous follow-ups [42].

It should be emphasized that in daily practice, the situation is probably even more difficult than that which can be assessed using data available from clinical trials. In clinical trial research, subjects usually receive only one topical medication whilst in everyday practice medications are often combined and this may affect the elevation of IOP of glaucoma patients and this result is supported by the previous study [42]. Hence, a combination treatment medication may affect the progression of glaucoma disease.

In conclusion, different individuals had different elevations of IOP and this leads that the progression of glaucoma disease for different individuals was different. Hence, the progression glaucoma differs from individual to individual because of different factors. The factors consist of socio-demographic characteristics, individual behaviors, and clinical factors. About 20% of the participants had elevated IOP in the study area. The elevation of IOP was high for rural glaucoma patients, aged patients, glaucoma patients with abnormal blood pressure, diabetic patients, glaucoma patients who are smoking and drinking alcohol, patients who had cataract surgery, and patients with trachoma disease.

Glaucoma may affect a person’s ability to drive and may mean their license to do so is withdrawn. Cataract surgeons and refractive options should be carefully conducted for glaucoma patients. For individuals with early glaucoma, all available modalities provide considerable advantages with little modification.

Health education related to potential risks of glaucoma with an elevated IOP on glaucoma patients, especially patients with abnormal blood pressure and diabetic patients should be conducted for patients to be cured easily of the disease. Proper treatment for glaucoma patients with additional ocular problems has many advantages including functional vision improvement, reduction of cost and efforts, and good safety.

The strength of this study lies in the fact that it suggests an interaction effect between certain characteristics of patients which did not exist in previous literature and is advantageous for future researchers to work in the area. Besides, the research tried to identify certain groups that require special attention and this helps to intervene and for the program to be effective.

This study is not without limitations. One limitation was that the effects of the significant interaction that existed in the study were observed during data analysis and were not considered in data collection. Therefore, how and why interaction effects of certain covariates affect the elevation of IOP level cannot be answered in this study. This can be considered as one potential research gap for future studies. The data had been taken in one treatment site, considering two or more treatment sites may have results different from these or may have additional information for these results. Hence, the need for further investigation including other treatment sites and additional predictor variables can be considered as potential research gaps for future investigators.

## Supplementary Information

**Additional file 1.**

## Data Availability

The data used for the current investigation is available in hands of the corresponding author. For further information, the data used under current investigation are submitted to the journal as supplementary material.

## Abbreviations

IOP: Intraocular pressure
LMM: Linear mixed model
AIC: Akaike Information Criterion
BIC: Bayesian information Criterion
